# The role of subjective, interpersonal, and structural social isolation in 12-month and lifetime anxiety disorders

**DOI:** 10.1186/s12889-024-18233-2

**Published:** 2024-03-11

**Authors:** Ann W. Nguyen, Harry Owen Taylor, Robert Joseph Taylor, Alexis Z. Ambroise, Tyrone Hamler, Weidi Qin, Linda M. Chatters

**Affiliations:** 1 Jack, Joseph and Morton Mandel School of Applied Social Sciences, Case Western Reserve University, 10900 Euclid Ave, 44106 Cleveland, OH USA; 2 Factor-Inwentash Faculty of Social Work, University of Toronto, 246 Bloor St W, M5S 1V4 Toronto, ON Canada; 3 School of Social Work, University of Michigan, 1080 S. University Ave, 48109 Ann Arbor, MI USA; 4 College of Education and Human Development, University of Delaware, 19716 Newark, DE USA; 5 Graduate School of Social Work, University of Denver, 2148 S. High Street, 80210 Denver, CO USA; 6 Sandra Rosenbaum School of Social Work, University of Wisconsin–Madison, 1350 University Ave, Madison, WI USA; 7 School of Public Health, University of Michigan, 1415 Washington Heights, 48109 Ann Arbor, MI USA

**Keywords:** Social isolation, Mental health, Anxiety, Psychiatric disorders, Social relationships

## Abstract

**Background:**

Anxiety disorders are among the most prevalent psychiatric conditions worldwide, and the incidence of anxiety disorders among adults in the U.S. have increased over the last decade. Anxiety disorders can have debilitating effects on multiple areas of functioning and quality of life. Recently, social isolation has emerged as an important public health problem associated with worse health and well-being outcomes. Research on the connection between social isolation and mental health has found that multiple dimensions of social isolation may negatively impact mental health, but few inquiries have focused on the association between social isolation and anxiety. This study examined the relationships between multiple dimensions of social isolation and anxiety disorders in a nationally representative sample of adults aged 18 and older.

**Methods:**

The sample includes 6082 individuals from the National Survey of American Life. This study examined whether three different dimensions of social isolation—subjective, interpersonal, and structural—were associated with 12-month and lifetime anxiety disorders (any anxiety disorder, posttraumatic stress disorder (PTSD), generalized anxiety disorder (GAD), panic disorder (PD), social anxiety disorder (SAD), and agoraphobia (AG). Logistic regressions were used to test the associations between the three social isolation variables and the anxiety outcomes.

**Results:**

This study found that of the three dimensions of social isolation, subjective isolation was most consistently related to both lifetime and 12-month anxiety disorders. Those who were subjectively isolated had increased odds of meeting criteria for any anxiety disorder, PTSD, GAD, PD, and AG over the past 12 months and throughout their lifetimes. Structural isolation was negatively associated with lifetime and 12-month AG.

**Conclusions:**

Public health approaches should include mental health and primary care providers and need to target social isolation, especially subjective isolation, which may be key in preventing anxiety disorders and the worsening of anxiety disorders. Future public health research is needed on how and in what ways the differing dimensions of social isolation impact mental health.

## Introduction

One in three adults have experienced an anxiety disorder in their lifetime, making anxiety disorders one of the most prevalent psychiatric problems worldwide [1, 2]. This prevalence may be on the rise, as the incidence of anxiety disorders among adults in the U.S. has increased over the last decade, especially among adults under age 50 [3]. Social anxiety disorder (SAD) is especially ubiquitous with a 13% rate of lifetime prevalence, followed by generalized anxiety disorder (GAD; 6.2% lifetime prevalence), panic disorder (PD; 5.2% lifetime prevalence), post-traumatic stress disorder (PTSD; 3.4% lifetime prevalence) [4], and agoraphobia (AG; 2.6% lifetime prevalence) [5].

Anxiety disorders can have debilitating effects on social, occupational, and other areas of functioning and quality of life for the individuals living with these conditions [6] and can even create financial burden [7]. Diagnoses of anxiety disorders are often comorbid with other psychiatric disorders, such as major depressive disorder, alcohol use disorder, and substance use disorder [8–10]. Anxiety disorders are also comorbid with physical health conditions, including asthma, back pain, arthritis, migraines, lung and heart disease, allergies, and ulcers [11, 12]. Moreover, adults living with an anxiety disorder are at higher risk for developing hypertension [13, 14] and disability [15].

Anxiety disorders have an economic cost to society as well. The financial burden of diseases and illnesses can be direct (medical and non-medical expenses resulting from the disease) or indirect (value of the loss of productivity caused by the disease), and anxiety is costly in both capacities. Inclusive of costs for treatment, medical expenses, lost productivity in the workforce, lost potential earnings, long-term opportunity costs, and costs associated with comorbid conditions, anxiety disorders are estimated to cost over $100 billion per year [16]. A better understanding of anxiety disorders and their risk factors would therefore be beneficial not only to the individuals living with these conditions, but also to society overall. One modifiable risk factor for anxiety that research identifies is social isolation. The current study will investigate how multiple dimensions of social isolation are associated with a range of lifetime and 12-month anxiety disorders in a nationally representative sample of adults.

### Importance of social connections over the life course

Social connections play a foundational role in human development and well-being at every stage of life. The convoy model of social relations [17] conceptualizes social support networks as convoys of social ties that follow a person throughout their life. Convoys of social relations change as the person moves through various stages of development and major life events and milestones (e.g., marriage, birth of a child, retirement). For example, convoys are expected to change in size and composition over the life course, adapting to the person’s needs and goals at each developmental stage and life event. Further, the nature of these relationships (e.g., frequency of contact and emotional closeness) are also expected to change over the life course.

This conceptualization of social relationships aligns well with the socioemotional selectivity theory [18, 19], which explains why people’s social support networks change over the life course. This theory posits that as a person ages, their perspective of their remaining time changes, and their social relational goals shift accordingly. As a person ages and perceives that their remaining time is contracting, they tend to prioritize high quality and emotional close relationships. Consequently, more distant relationships, such as relationships with acquaintances, are less likely to be maintained, and this results in a reduction in the size of the person’s support network. However, as the person’s support network decreases in size, the overall quality of their social ties tends to increase [20].

Given their prominence and function, social connections have important implications for mental health. A systematic review of the literature on social relationships and depression found that social support and support network size and diversity were the strongest and most consistent predictors of depression [21]; that is, people who received more emotional and instrumental support, had larger support networks, and had support networks with greater role diversity were less likely to experience depression. Another systematic review indicated that people who received more social support reported decreased depression severity, less severe anxiety symptoms, and higher rates of remission from an anxiety disorder [22]. In addition to affective problems, social ties play an important role in cognitive function. For instance, in Crooks et al.’s [23] longitudinal study, they found that among older women who were free of dementia at baseline, women who had smaller support networks were more likely to experience the onset of dementia at follow-up than women who had larger support networks. Overall, this body of empirical evidence underscores the importance of social connections for mental health.

### Social isolation and mental health

Given the critical impact that social connections have on mental health, the rapid increase in social isolation presents a particularly pressing concern for public health [24]. The ubiquity of social isolation in our society is so great that the U.S. Surgeon General recently declared social isolation and loneliness (a subjective form of social isolation) an epidemic [25]. Social isolation is consistently associated with worse health and well-being outcomes. Previous studies find that social isolation is associated with earlier mortality, worse self-rated health, worse cardiovascular health, depressive symptoms, psychological distress, and cognitive decline [26]. Furthermore, objective social isolation is associated with increased Medicare spending among older adults [27]. Given its deleterious health effects, numerous government and philanthropic organizations around the world are actively working to mitigate social isolation [28–30].

The detrimental effects of social isolation on mental health are well-documented. In fact, in the last few years, the National Academies of Sciences, Engineering and Medicine and the U.S. Surgeon General both issued separate yet comprehensive reports on the health effects of social isolation. These reports identified a broad range of mental health problems associated with social isolation in the general population. Research in this area demonstrates that people who are more lonely have a higher likelihood of reporting clinically significant depression, anxiety, and suicidal ideation than people who are less lonely [25, 26]. Similarly, research specifically on older adults shows that people who report higher levels of loneliness/subjective isolation also report a greater number of depressive symptoms [31], more severe depressive symptoms [32], and higher levels of psychological distress [32]. These findings demonstrate the harmful effects of social isolation across the adult life span.

Social isolation has been operationalized in a multitude of ways. In the current study, we recognize that social isolation is a multidimensional concept and have operationalized social isolation as having three different dimensions: interpersonal, structural, and subjective social isolation [33]. Interpersonal and structural social isolation reflect an objective situation in which there is a tangible absence of social relationships. Interpersonal social isolation has previously been operationalized as infrequent contact with social support network members. Structural isolation, on the other hand, has been operationalized as the number of children an individual has and the number of persons in their household [33]. Subjective isolation, on the other hand, has been operationalized as the lack of perceived emotional closeness to people in one’s social network [32, 34, 35].

Research on the connection between social isolation and mental health finds that multiple dimensions of social isolation negatively impact mental health and well-being. For instance, Taylor et al.’s study on social isolation in adults found that people who were objectively isolated and subjectively isolated reported higher levels of depressive symptoms and psychological distress compared to people who were not objectively or subjectively isolated [35, 36]. In another study, Nguyen et al. found that subjective social isolation predicted increased risk for major depressive disorder, any 12-month DSM-IV disorder, and a greater number of 12-month disorders [34]. However, their study did not find statistically significant relationships between objective isolation and psychiatric problems. A more recent study used a nationally representative sample of older Black adults from the Health and Retirement Study to examine the interrelations between (objective) social isolation, loneliness, and mental health [37]. This study indicated that older Black adults who were lonely were more likely to have met criteria for a psychiatric disorder within their lifetimes and reported more depressive symptoms than their non-lonely counterparts [37]. In contrast, objective social isolation did not predict either mental health outcomes in this study [37]. Another study that examined the effects of loneliness in a nationally representative sample of adults in mid- and late life found that middle-aged and older adults who were more lonely also reported more depressive symptoms than their less lonely peers [31].

These studies underscore the pernicious and complex effects of social isolation on mental health. More importantly, the findings from these studies indicate that dimensions of social isolation do not function similarly in relation to mental health. This argues for a more nuanced investigation of the relationship between social isolation and mental health, in which the diverse dimensions of social isolation are parsed out to understand how they uniquely influence mental health. While there are numerous studies on the mental health effects of social isolation, most research in this area is focused on depression, depressive symptoms, and psychological distress [34, 35, 37]. Thus, the extant studies are limited in the range of mental health problems examined. For instance, very few studies focused on the entire adult life span examine how social isolation relates to anxiety [38], which is distinct from depression and psychological distress. Of the studies that examine anxiety, many focus on anxiety symptoms rather than specific diagnosable anxiety disorders, which hold greater clinical significance. A better understanding of how anxiety disorders are related to social isolation is imperative, as these are some of the most common mental health problems in the U.S. that can lead to devastating social, functional, and economic impairments.

### Purpose of the present study

The current study aims to address the dearth of knowledge regarding the role of social isolation in anxiety disorders by examining the relationships between multiple dimensions of social isolation and anxiety disorders in a nationally representative sample of community-dwelling adults aged 18 and older. Specifically, the aim of this study is to determine whether three different dimensions of social isolation—subjective, interpersonal, and structural—are associated with 12-month and lifetime anxiety disorders (any anxiety disorder, PTSD, GAD, SAD, PD, AG).

## Methods

### Data

The National Survey of American Life: Coping with Stress in the 21st Century (NSAL) was collected by the Institute of Social Research’s Survey Research Center, in cooperation with the Program for Research on Black Americans [39]. The NSAL is a national psychiatric epidemiological survey focused on African American and Black Caribbean adults in the U.S. The data collection was conducted from February 2001 to June 2003; the overall response rate was 72.3%. Most of the interviews were conducted face-to-face (86%) in respondents’ homes, while the remaining 14% were telephone interviews. A total of 6,082 face-to-face interviews were conducted with persons aged 18 or older, including 3,570 African American respondents, 891 non-Latinx White respondents, and 1,621 Black respondents of Caribbean descent. The NSAL was approved by the University of Michigan Institutional Review Board. The NSAL is available from the Inter-University Consortium for Political and Social Research’s website (10.3886/ICPSR20240.v8).

### Dependent variables

There are 12 dependent variables in this analysis representing the prevalence of both 12-month and lifetime: (1) any anxiety disorder, (2) posttraumatic stress disorder (PTSD), (3) generalized anxiety disorder (GAD), (4) panic disorder (PD), (5) social anxiety disorder (SAD), and (6) agoraphobia (AG). Assessment of any anxiety disorder (lifetime and 12-month) included these 6 disorders: PTSD, GAD, PD, SAD, AG, and obsessive-compulsive disorder (OCD). All anxiety variables except obsessive-compulsive disorder were assessed using the Diagnostic and Statistical Manual of Mental Disorders, Fourth Edition (DSM-IV) World Mental Health Composite International Diagnostic Interview (WMH-CIDI) [40]. OCD was assessed using the CIDI short-form version (CIDI-SF).

### Independent variables

There are three main independent variables in this analysis: (1) subjective isolation (2) interpersonal isolation, and (3) structural isolation. Subjective isolation was measured by the sum of subjective isolation from family and from friends using the question, “How close do you feel towards your family members? Would you say very close, fairly close, not too close, or not close at all?” Not too close and not close at all were coded 1, and all other responses were coded 0. Subjective isolation from friends was assessed in the same manner as subjective isolation from family and was coded in the same manner (not too close and not close at all = 1; all other = 0). Scores for the subjective isolation variable ranged from 0 to 2, with higher scores indicating greater levels of subjective isolation.

Interpersonal social isolation was measured by summing the following six measures: (1) being unmarried and having no romantic involvement, and reported isolation from: (2) neighbors, (3) neighborhood groups, (4) congregational members, (5) family members, and (6) friends. Marital/romantic status was assessed by combining two items. First, respondents were asked if they are currently: married, living with a partner, separated, divorced, widowed, or never married. Previously married (separated, divorced, widowed) and never married respondents were additionally asked whether they were currently involved in a romantic relationship. Respondents who were unmarried/no romantic involvement were coded 0; those who were married, cohabiting or had a main romantic involvement were coded 1. Isolation from neighbors was assessed by the question: “How often do you get together with any of your neighbors, that is, either visiting each other’s homes or going places together? Would you say nearly every day, at least once a week, a few times a month, at least once a month, a few times a year or never?” A few times a year and never were coded 1, and all other responses were coded 0. Isolation from neighborhood groups was assessed with two questions: “Are there any groups in this neighborhood such as block clubs, community associations, social clubs, helping groups and so forth?” If respondents answered yes, they were then asked, “Are you involved with any of these groups?” Not being involved with a neighborhood group was coded 1, and all other responses were coded 0. Isolation from congregation members was assessed by the item: “How often do you see, write, or talk on the telephone with members of your church (place of worship)? Would you say nearly every day, at least once a week, a few times a month, at least once a month, a few times a year or never?” A few times a year, never, and those who never attend religious services were coded 1, and all other responses were coded 0 (consistent with research in this field, the contact with congregation member question was asked only of respondents who indicated that they attend religious services at least a few times a year). Isolation from family was assessed by the item: “How often do you see, write or talk on the telephone with family or relatives who do not live with you? Would you say nearly everyday, at least once a week, a few times a month, at least once a month, a few times a year, hardly ever or never?” A few times a year and never were coded 1; all other responses were coded 0. Isolation from friends was assessed using the same methods as isolation from family. Scores for the interpersonal isolation variable ranged from 0 to 6, with higher scores indicating greater levels of interpersonal isolation.

Structural social isolation was measured by the sum of two items: (a) being childless and (b) living alone. Living alone was coded 1 and living with others was coded 0; similarly, being childless was coded 1 and having a living child was coded 0. Scores for the structural isolation variable ranged from 0 to 2, with higher scores indicating greater levels of structural isolation.

### Covariates

Covariates for the study included race/ethnicity, gender, age (in years), education (in years), household income (in U.S. dollars). Race/ethnicity differentiated between non-Latinx White, African American, and Black Caribbean respondents. Gender differentiated between female and male. Age, years of education, and household income were measured continuously.

### Analysis strategy

We used multivariable logistic regression to test the associations between the three social isolation variables and the anxiety outcomes. Multivariable logistic regression was most suitable for exploring the objectives of this study, as we sought to determine the possibly predictive relationship between social isolation and binary categorical outcome variables pertaining to anxiety. For each anxiety outcome, we conducted four logistic regression models. The first model regressed the anxiety outcome on subjective isolation only; the second model regressed the anxiety outcome on interpersonal isolation only; the third model regressed the anxiety outcome on structural isolation only; and the final model regressed the anxiety outcome on all three social isolation measures. Furthermore, all models controlled for all covariates.

All analyses were conducted using Stata, which uses the Taylor expansion approximation technique for calculating the complex design-based estimates of variance. All statistical analyses accounted for the complex multistage clustered design of the NSAL sample, unequal probabilities of selection, nonresponse, and poststratification to calculate weighted, nationally representative population estimates and standard errors.

## Results

The distribution of the study variables is presented in Table 1. The vast majority of respondents were not socially isolated with regards to subjective, interpersonal, or structural isolation. Rates for 12-month and lifetime anxiety disorders were consistent with prior psychiatric epidemiological research [41]. The most common 12-month and lifetime anxiety disorder reported among the sample was PTSD. Given the focus of the NSAL, the sample is weighted to be roughly half non-Latinx white and half Black American (African American and Black Caribbean). There were more women (54.13%) in the sample than men. The average age of respondents was 43 years. The mean household income was $42,417, and respondents had an average of 12.89 years of formal education.

**Table 1:** Demographic Characteristics of the Sample and Distribution of Study Variables

	% (M)	N (S.D.)
12-month Any Anxiety Disorder	11.19	5001 (0.24)
12-month Posttraumatic Stress Disorder	3.89	4998 (0.15)
12-month Generalized Anxiety Disorder	2.78	5884 (0.16)
12-month Panic Disorder	2.70	5885 (0.16)
12-month Social Anxiety Disorder	5.69	5009 (0.06)
12-month Agoraphobia	1.19	5886 (0.11)
Lifetime Any Anxiety Disorder	19.61	5002 (0.30)
Lifetime Posttraumatic Stress Disorder	9.05	4998 (0.22)
Lifetime Generalized Anxiety Disorder	5.90	5884 (0.24)
Lifetime Panic Disorder	4.72	5885 (0.21)
Lifetime Social Anxiety Disorder	9.82	5009 (0.16)
Lifetime Agoraphobia	2.15	5886 (0.15)
Subjective Isolation	0.19	6065 (0.44)
Interpersonal Isolation	2.00	6066 (1.03)
Structural Isolation	0.50	6082 (0.65)
**Race/Ethnicity**		
African American	46.82	3570
Black Caribbean	3.51	1621
Non-Latinx White	49.67	891
**Gender**		
Male	45.87	2286
Female	54.13	3796
Age	43.57	6082 (16.61)
Household Income	42417.66	6082 (39411.54)
Education	12.89	6082 (2.65)

Percents are weighted; frequencies are unweighted. M = Mean, S.D. = Standard Deviation

Percents and N’s are presented for categorical variables; Means and Standard Deviations are presented for continuous variables

Table 2 presents the results of the multivariable logistic regression analyses of the relationships between the three social isolation variables and 12-month anxiety disorders. Subjective isolation was significantly and positively associated with meeting criteria for PTSD, GAD, PD, AG, and any disorder, indicating that adults who were subjectively isolated had a higher likelihood of having an anxiety disorder. Structural isolation was negatively associated with 12-month AG, indicating that adults who experienced increased structural isolation were less likely to have AG. Neither subjective, interpersonal, nor structural social isolation were significantly associated with 12-month SAD.

**Table 2:** Logistic regression analyses of 12-month anxiety and social isolation

	Model 1	Model 2	Model 3	Model 4
	OR(95% CI)	OR(95% CI)	OR(95% CI)	OR(95% CI)
**Any anxiety disorder**				
Subjective isolation	1.72 (1.44, 2.05)***	–	–	1.72 (1.42,2.09)***
Interpersonal isolation	–	1.07 (0.94,1.21)	–	0.99 (0.87,1.14)
Structural isolation	–	–	0.94 (0.77,1.15)	0.95 (0.77,1.16)
**Posttraumatic stress disorder**				
Subjective isolation	1.56 (1.08, 2.28)*	–	–	1.49 (1.02,2.18)*
Interpersonal isolation	–	1.14 (0.93,1.41)	–	1.09 (0.89,1.33)
Structural isolation	–	–	0.82 (0.59,1.13)	0.82 (0.60,1.13)
**Generalized anxiety disorder**				
Subjective isolation	2.03 (1.34, 3.09)**	–	–	1.79 (1.16,2.77)**
Interpersonal isolation	–	1.35 (1.02,1.78)*	–	1.23 (0.93,1.61)
Structural isolation	–	–	1.04 (0.59,1.84)	1.03 (0.59,1.79)
**Panic disorder**				
Subjective isolation	1.97 (1.19, 3.28)**	–	–	2.04 (1.21,3.44)**
Interpersonal isolation	–	1.04 (0.84,1.30)	–	0.94 (0.77,1.16)
Structural isolation	–	–	0.94 (0.67,1.31)	0.96 (0.69,1.34)
**Social anxiety disorder**				
Subjective isolation	1.37 (0.39,4.80)	–	–	1.20 (0.41,3.52)
Interpersonal isolation	–	1.37 (0.73,2.58)	–	1.34 (0.80,2.23)
Structural isolation	–	–	1.18 (0.55,2.50)	1.18 (0.54,2.58)
**Agoraphobia**				
Subjective isolation	1.67 (1.08,2.57)*	–	–	1.74 (1.17,2.61)**
Interpersonal isolation	–	0.92 (0.63,1.36)	–	0.86 (0.61,1.23)
Structural isolation	–	–	0.51 (0.28,0.92)*	0.52 (0.29,0.91)*

IRR = Incident Rate Ratio; 95%CI = 95% Confidence Interval

Note: Significance test of the individual parameter estimates was based on a complex design-corrected *t*-test ^4^Race/ethnicity, age, gender, household income, education, were controlled in all models

The analysis of lifetime anxiety disorders and the three social isolation variables are presented in Table 3. A comparison of Model 4 (full model) in the analysis of lifetime anxiety disorders (Table 3) and 12-month anxiety disorders (Table 2) demonstrated that, in terms of significance and relationship direction, the results were identical.

**Table 3:** Logistic regression analyses of lifetime anxiety and social isolation

	Model 1	Model 2	Model 3	Model 4
	OR(95% CI)	OR(95% CI)	OR(95% CI)	OR(95% CI)
**Any anxiety disorder**				
Subjective isolation	1.56 (1.33, 1.84)***	–	–	1.55 (1.31,1.83)***
Interpersonal isolation	–	1.07 (0.98,1.16)	–	1.01 (0.93,1.10)
Structural isolation	–	–	0.92 (0.78,1.08)	0.92 (0.79,1.08)
**Posttraumatic stress disorder**				
Subjective isolation	1.47 (1.16, 1.85)**	–	–	1.47 (1.18,1.84)**
Interpersonal isolation	–	1.03 (0.93,1.15)	–	0.99 (0.89,1.09)
Structural isolation	–	–	0.83 (0.68,1.02)	0.84 (0.69,1.03)
**Generalized anxiety disorder**				
Subjective isolation	1.56 (1.14, 2.14)**	–	–	1.51 (1.09,2.10)*
Interpersonal isolation	–	1.13 (0.99,1.30)	–	1.07 (0.93,1.22)
Structural Isolation	–	–	1.23 (0.80,1.91)	1.24 (0.80,1.92)
**Panic disorder**				
Subjective isolation	1.79 (1.26, 2.55)**	–	–	1.92 (1.32,2.79)***
Interpersonal isolation	–	0.95 (0.75,1.20)	–	0.89 (0.70,1.12)
Structural isolation	–	–	0.74 (0.54,1.03)	0.77 (0.56,1.05)
**Social anxiety disorder**				
Subjective isolation	1.08 (0.72,1.63)	–	–	1.11 (0.74,1.66)
Interpersonal isolation	–	0.96 (0.79,1.16)	--	0.95 (0.79,1.14)
Structural isolation	–	–	0.98 (0.76,1.26)	0.99 (0.77,1.26)
**Agoraphobia**				
Subjective isolation	1.35 (1.02,1.78)*	–	–	1.44 (1.00,2.08)*
Interpersonal isolation	–	0.87 (0.54,1.41)	–	0.85 (0.52,1.37)
Structural isolation	–	–	0.56 (0.31,1.00)	0.57 (0.34,0.97)*

IRR = Incident Rate Ratio; 95%CI = 95% Confidence Interval

Note: Significance test of the individual parameter estimates were based on a complex design-corrected *t*-test

^4^Race/ethnicity, age, gender, household income, education, were controlled in all models

Contrary to expectations, respondents who were structurally isolated had a lower likelihood of having either 12-month or lifetime AG. To better understand these relationships, we conducted additional supplemental analyses by examining the impact of the two indicators of structural isolation separately. We found that living alone was unrelated to both 12-month and lifetime AG. Being childless, however, was negatively associated with AG. Adults who did not have any children were less likely to have either 12-month or lifetime AG. This finding remained consistent with or without controlling for subjective and interpersonal isolation.

## Discussion

The current analysis investigated the relationship between interpersonal, structural, and subjective social isolation and 12-month and lifetime anxiety disorders in a nationally representative sample of community-dwelling adults. This study is the first to simultaneously examine how multiple dimensions of social isolation are related to a range of DSM-IV anxiety disorders in a nationally representative sample of Black and white Americans aged 18 and older. The findings indicate that of the three dimensions of social isolation, subjective isolation was most consistent in its relationship with both lifetime and 12-month anxiety disorders. Specifically, people who were subjectively isolated had increased odds of meeting criteria for any anxiety disorder, PTSD, GAD, PD, and AG over the past 12 months and at some point during their lifetimes.

These findings are concordant with prior research demonstrating that subjective isolation is predictive of mental health problems. Empirical studies indicate that people who are subjectively isolated report more depressive symptoms [31, 32, 35, 37] and psychological distress [35]. Additionally, subjective isolation is associated with increased odds for any lifetime psychiatric disorder [37] and a greater number of 12-month psychiatric disorders [34]. The consistent associations between subjective isolation and anxiety disorders identified in this study are also concordant with some evidence indicating that subjective isolation, in particular, is a stronger and more reliable predictor of mental health problems than other dimensions of isolation, such as structural isolation [34]. For example, Nguyen et al.’s research found that people who were objectively isolated were more likely to experience depression [34]. However, when controls for subjective isolation were included in the model, objective isolation no longer predicted depression, and instead, subjective isolation was significantly associated with an increased risk for depression. Similarly, Taylor et al. found that people who were subjectively isolated reported higher levels of psychological distress than people who were not subjectively isolated [32]. Yet, in their study, the level of psychological distress did not differ between people who were objectively isolated and people who were not objectively isolated. In other words, their study found that subjective isolation influenced distress, while objective isolation did not. Together, this prior evidence and this study’s findings suggest that perceptions and appraisals of relational quality and affective relational features—especially emotional closeness to support network members or lack thereof—may have a more significant impact on mental health than more objective dimensions of social isolation (i.e., structural and interpersonal).

The current findings underscore the detrimental effects of social isolation on mental health. Social relationships can influence mental health in several ways. Social ties, especially those that are emotionally close, can create a sense of meaning and purpose, which, in turn, can lead to improved emotion regulation [25, 26]. Social relationships are also important in the stress process because they can influence stress appraisal and coping strategies [25, 26]. That is, people who are socially integrated have more social resources for dealing with life stressors, so they tend to appraise potentially problematic situations as less stressful. Additionally, when these individuals experience stress, their social connections can serve as stress coping resources, and thus minimize the stress response. Social connections can also impact mental health through social influence [25, 26]. Shared social norms within a person’s support network and support and encouragement from network members can positively shape a person’s health behaviors (e.g., diet and physical activity), which can then lead to mental health and well-being. All these processes are important in protecting against mental health problems.

It is important to note that the relationship between social isolation and anxiety disorders is bidirectional. Social avoidance and withdrawal is a well-documented feature of a number of anxiety disorders [6]. That is, people who are living with an anxiety disorder are more likely to withdraw from their support networks and avoid social situations, and these socially avoidant behaviors can reinforce their anxiety and worsen the anxiety disorder. Research also demonstrates that people who are socially isolated are at greater risk for developing an anxiety disorder. Prospective studies show that among people without an anxiety disorder, social isolation, particularly subjective isolation, can lead to the onset of new mental health problems [42]. For example, a systematic review of research on loneliness and mental health identified numerous prospective American and international studies that found that people who were lonely at baseline were more likely to experience the onset of anxiety or depression at later waves [42]. Further, several of these studies excluded participants who currently or previously had depression or anxiety, ensuring that depression or anxiety identified in subsequent waves were new onsets. Importantly, several studies in this area controlled for objective isolation and other measures of social integration. The prospective study design and rigorous modeling strategies of these studies strongly indicate that subjective isolation can lead to the onset of anxiety and other mental health problems over time.

Interestingly, our findings revealed that structural isolation was associated with decreased likelihood for lifetime and 12-month AG. These findings contradicted our expectations, leading us to conduct ancillary analyses (not shown) to better understand them. In the ancillary analyses, we decomposed the effects of structural isolation, and the analyses indicated that childlessness was driving these associations. More specifically, the analyses demonstrated that while living alone was unassociated with 12-month and lifetime AG, being childless was associated with decreased odds for 12-month and lifetime AG. While we were unable to identify prior research with similar findings, this is likely because most social isolation studies do not parse out the varying dimensions of social isolation in the same manner as our analysis. One possible explanation for the current findings is that people who were childless may have fewer close sources of support that would enable them to engage in avoidant behaviors that could contribute to and reinforce their anxiety. That is, people without children, especially adult children, may be less likely to engage in avoidant behaviors (e.g., avoiding the grocery store, not leaving home). because they do not have children who can enable these avoidant behaviors (e.g., child bringing groceries). Thus, this decreased ability to engage in avoidant behaviors may decrease their functional impairments and their risk for AG. Moreover, people who struggle with anxiety and have children may be more motivated to seek help for their mental health problem, so that they can appropriately care for their children.

There are several study limitations that should be noted. First, the data were cross-sectional, so conclusions regarding causal direction are not possible. The temporal sequence of social isolation and anxiety disorder cannot be established. Thus, it remains unclear whether people in this study who were subjectively isolated were more likely to develop anxiety disorder, or if people who experienced an anxiety disorder were more likely to perceive their relationships to be more emotionally distant (i.e., subjectively isolated). As we noted previously, it is likely that the relationship between social isolation and anxiety disorders are *bi-directional and synergistic*, which is supported by strong evidence. Nevertheless, we recommend that future research use longitudinal methodologies to determine the temporal sequence of social isolation and anxiety disorders to better understand the causal relationship between these two variables. Second, the NSAL was collected in 2001–2003, which limits the generalizability of the findings to the current population and contemporary relationships. Despite this, the NSAL is still the only national psychiatric epidemiological data set with both extensive social support network information and a relatively large and diverse Black sample (African Americans and Black Caribbeans). Third, the NSAL did not sample institutionalized and non-community dwelling people; as a result, the findings from this study cannot be generalized to these populations, and all generalizations extend only to community-dwelling adults.

Despite these limitations, this study has several notable strengths. This is the first study to simultaneously examine multiple dimensions of social isolation in relation to a broad range of diagnosable anxiety disorders. Our study contributes to the literature by parsing out the differing social isolation dimensions, which allowed us to identify their unique associations with anxiety disorders. Our findings suggest that future research should disaggregate the varying dimensions of social isolation rather than treating it as a unidimensional concept. This can foster the development of a more complete understanding of how different aspects or manifestations of social isolation influence mental health. Another strength of this study is the use of a national probability sample of adults, which permits for generalization of the study findings to the population level (of community-dwelling Black and white American adults). The focus on DSM-IV anxiety disorders is an additional strength of the study. While most of the research on the connection between social isolation and mental health throughout the adult life span focuses predominantly on depressive symptoms, depression, and psychological distress, far fewer studies examine how social isolation impacts anxiety. Of the few studies on anxiety in adults, most use anxiety scales or symptoms checklists to assess anxiety, which are limited in their clinical relevance. In contrast, this study assessed specific DSM-IV anxiety disorders derived from the WMH-CIDI, which is a fully structured diagnostic interview with excellent inter-rater reliability, good test-retest reliability, and good validity [43].

### Public health implications and conclusions

The current findings indicate that subjective isolation is consistently associated with increased odds for a range of anxiety disorders, and they suggest that being emotionally distant from support network members may be a risk factor for some anxiety disorders. Public health approaches that target reductions in social isolation, especially subjective isolation, are important for preventing anxiety disorders and the worsening of anxiety disorders. Further, both mental health care specialists and primary care providers are encouraged to assess patients’ social ecology and screen for social isolation. Patients who are identified to be socially isolated or at high risk of social isolation should be offered or referred out for support, resources, and/or intervention to increase social connections and integration. Finally, more public health research is necessary to better understand how and in what ways the differing dimensions of social isolation impact mental health. In particular, research in this area on minoritized populations is severely limited. Minoritized people are at greater risk for social isolation given their marginalized status at multiple overlapping and intersecting levels in society. Focused research on social isolation specifically in minoritized groups will not only address this knowledge gap but will also clarify unique risk factors for social isolation in these groups and how social isolation relates to mental health in these communities.

In conclusion, this study demonstrated the detriments of social isolation within the context of several anxiety disorders and revealed that different dimensions of social isolation have varying associations with specific anxiety disorders. The differing relationships between subjective and structural isolation argue for a more nuanced approach to investigating the effects of social isolation on mental health.

## Data Availability

The dataset is available from the Inter-University Consortium for Political and Social Research’s website (10.3886/ICPSR20240.v8).

## References

[1] Bandelow B, Michaelis S (2022). Epidemiology of anxiety disorders in the 21st century. Dialog Clin Neurosci.

[2] Szuhany KL, Simon NM (2022). Anxiety disorders: a review. JAMA.

[3] Goodwin RD, Weinberger AH, Kim JH, Wu M, Galea S (2020). Trends in anxiety among adults in the United States, 2008–2018: Rapid increases among young adults. J Psychiatr Res.

[4] Schein J, Houle C, Urganus A, Cloutier M, Patterson-Lomba O, Wang Y (2021). Prevalence of post-traumatic stress disorder in the United States: a systematic literature review. Curr Med Res Opin.

[5] Kessler RC, Petukhova M, Sampson NA, Zaslavsky AM, Wittchen HU (2012). Twelve-month and lifetime prevalence and lifetime morbid risk of anxiety and mood disorders in the United States. Int J Methods Psychiatr Res.

[6] American Psychiatric Association (2013). Diagnostic and statistical manual of mental disorders.

[7] Hoffman DL, Dukes EM, Wittchen HU (2008). Human and economic burden of generalized anxiety disorder. Depress Anxiety.

[8] Brady KT, McCauley JL, Back SE. The comorbidity of post-traumatic stress disorder (PTSD) and substance use disorders. Textbook Addict Treatment: Int Perspect. 2021:1327–39.

[9] Fox R, Hyland P, Power JM, Coogan AN (2020). Patterns of comorbidity associated with ICD-11 PTSD among older adults in the United States. Psychiatry Res.

[10] Marmorstein NR (2012). Anxiety disorders and substance use disorders: different associations by anxiety disorder. J Anxiety Disord.

[11] El-Gabalawy R, Mackenzie CS, Shooshtari S, Sareen J (2011). Comorbid physical health conditions and anxiety disorders: a population-based exploration of prevalence and health outcomes among older adults. Gen Hosp Psychiatry.

[12] Niles AN, Dour HJ, Stanton AL, Roy-Byrne PP, Stein MB, Sullivan G (2015). Anxiety and depressive symptoms and medical illness among adults with anxiety disorders. J Psychosom Res.

[13] Johnson HM (2019). Anxiety and hypertension: is there a link? A literature review of the comorbidity relationship between anxiety and hypertension. Curr Hypertens Rep.

[14] Lim L-F, Solmi M, Cortese S (2021). Association between anxiety and hypertension in adults: a systematic review and meta-analysis. Neurosci Biobehavioral Reviews.

[15] Dong L, Freedman VA, de Leon CFM (2020). The association of comorbid depression and anxiety symptoms with disability onset in older adults. Psychosom Med.

[16] Kessler RC, Greenberg PE. The economic burden of anxiety and stress disorders. Neuropsychopharmacology: The Fifth Generation of Progress. 2002;67:982– 92.

[17] Kahn RL, Antonucci TC, Baltes PB, Brim OG (1980). Convoys over the life course: attachment, roles, and social support. Life-span development and behavior.

[18] Carstensen LL, Isaacowitz DM, Charles ST (1999). Taking time seriously: a theory of socioemotional selectivity. Am Psychol.

[19] Carstensen LL, Carstensen LL, Edelstein BA (1987). Age-related changes in social activity. Handbook of clinical gerontology.

[20] Carstensen LL (1992). Social and emotional patterns in adulthood: support for socioemotional selectivity theory. Psychol Aging.

[21] Santini ZI, Koyanagi A, Tyrovolas S, Mason C, Haro JM (2015). The association between social relationships and depression: a systematic review. J Affect Disord.

[22] Wang J, Mann F, Lloyd-Evans B, Ma R, Johnson S (2018). Associations between loneliness and perceived social support and outcomes of mental health problems: a systematic review. BMC Psychiatry.

[23] Crooks VC, Lubben J, Petitti DB, Little D, Chiu V (2008). Social network, cognitive function, and dementia incidence among elderly women. Am J Public Health.

[24] Na PJ, Jeste DV, Pietrzak RH (2023). Social disconnection as a global behavioral epidemic—A call to action about a major health risk factor. JAMA Psychiatry.

[25] United States (2023). Public Health Service. Office of the Surgeon General. Our epidemic of loneliness and isolation: the U.S. Surgeon General’s advisory on the healing effects of social connection and community.

[26] National Academies of Sciences E, and Medicine (2020). Social isolation and loneliness in older adults: opportunities for the health care system.

[27] Shaw JG, Farid M, Noel-Miller C, Joseph N, Houser A, Asch SM (2017). Social isolation and Medicare spending: among older adults, objective isolation increases expenditures while loneliness does not. J Aging Health.

[28] Elder K, Retrum J (2012). Framework for isolation in adults over 50: AARP Foundation/isolation Framework Project.

[29] Global Initiative on Loneliness and Connection. Position statement on addressing social isolation, loneliness, and the power of human connection. 2022.

[30] Lubben J, Gironda M, Sabbath E, Kong J, Johnson C. Social isolation presents a grand challenge for social work. American Academy of Social Work and Social Welfare; 2015.

[31] Taylor HO, Nguyen AW. Depressive symptoms and loneliness among black and white older adults: the moderating effects of race. Innov Aging. 2020;4(5). 10.1093/geroni/igaa048 PMC7739884 33354628

[32] Taylor HO, Taylor RJ, Nguyen AW, Chatters LM (2018). Social isolation, depression, and psychological distress among older adults. J Aging Health.

[33] Taylor HO, Taylor RJ (2020). Interpersonal and structural social isolation among African American and Black Caribbean men. Int J Mens Social Community Health.

[34] Nguyen AW, Taylor RJ, Taylor HO, Chatters LM (2020). Objective and subjective social isolation and psychiatric disorders among African americans. Clin Soc Work J.

[35] Taylor RJ, Taylor HO, Nguyen AW, Chatters LM (2020). Social isolation from family and friends and mental health among African americans and Black Caribbeans. Am J Orthopsychiatry.

[36] Taylor RJ, Taylor HO, Chatters LM. Social isolation from extended family members and friends among African americans: findings from a national survey. J Family Social Work. 2016:1–19. 10.1080/10522158.2016.1181127 PMC5142749 27942198

[37] Taylor HO (2021). Social isolation, loneliness, and physical and mental health among Black older adults. Annual Rev Gerontol Geriatr.

[38] Park C, Majeed A, Gill H, Tamura J, Ho RC, Mansur RB (2020). The effect of loneliness on distinct health outcomes: a comprehensive review and meta-analysis. Psychiatry Res.

[39] Jackson JS, Torres M, Caldwell CH, Neighbors HW, Nesse RM, Taylor RJ (2004). The National Survey of American Life: a study of racial, ethnic and cultural influences on mental disorders and mental health. Int J Methods Psychiatr Res.

[40] Kessler RC, Ustün TB (2004). The world mental health (WMH) survey initiative version of the world health organization (WHO) composite international diagnostic interview (CIDI). Int J Methods Psychiatr Res.

[41] Vilsaint CL, NeMoyer A, Fillbrunn M, Sadikova E, Kessler RC, Sampson NA (2019). Racial/ethnic differences in 12-month prevalence and persistence of mood, anxiety, and substance use disorders: variation by nativity and socioeconomic status. Compr Psychiatr.

[42] Mann F, Wang J, Pearce E, Ma R, Schlief M, Lloyd-Evans B (2022). Loneliness and the onset of new mental health problems in the general population. Soc Psychiatry Psychiatr Epidemiol.

[43] Andrews G, Peters L (1998). The psychometric properties of the Composite International Diagnostic interview. Soc Psychiatry Psychiatr Epidemiol.

